# Nailbed HPV-related Bowen’s disease in a man living with HIV

**DOI:** 10.1177/09564624241234085

**Published:** 2024-02-20

**Authors:** Manik Kohli, Georgios Kravvas, Arthur Wong, David Zargaran, Peter Ellery, Christopher B Bunker

**Affiliations:** 1 Department of Sexual Health and HIV, 4954 Central and North West London NHS Foundation Trust, London, UK; 2 Institute for Global Health, 4919 University College London, London, UK; 3 Department of Dermatology, 8964 University College London Hospitals NHS Foundation Trust, London, UK; 4 Department of Plastic Surgery, 4965 Royal Free London NHS Foundation Trust, London, UK; 5 Department of Histopathology, 8964 University College London Hospitals NHS Foundation Trust, London, UK; 6 Department of Histopathology, Leeds Teaching Hospitals NHS Trust, Leeds, UK

**Keywords:** HPV (human papillomavirus) < viral disease, HIV (human immunodeficiency virus) < viral disease, vaccination < other, sexual behaviour < other, diagnosis < other

## Abstract

Human papillomavirus (HPV) is a common sexually transmitted infection with wide-ranging clinical manifestations. High-risk anogenital HPV genotypes have also been reported to cause extragenital disease. We describe the case of a 69-year-old male patient living with HIV who was diagnosed with HPV-16 associated Bowen’s Disease (BD) of the right middle finger nailbed, despite good virologic control and immune reconstitution. The lesion was managed surgically with adjunctive post-exposure HPV vaccination. This case adds to the growing body of evidence of extra-genital HPV disease attributable to anogenital genotypes in people living with HIV.

## Introduction

Human papillomavirus (HPV) is the most common sexually transmitted infection globally, and can cause a wide spectrum of clinical manifestations; ranging from benign to pre-malignant and malignant lesions.^1–3^ High-risk (HR) HPV genotypes 16 and 18 are among the most common HPVs detected and are a significant risk factor for malignant disease, typically at anogenital sites.^1,2,4^ People living with HIV are at greater risk of HPV-related disease.^2,5^ Despite the advent of antiretroviral treatment (ART), a lower nadir CD4 count has been associated with severe HPV-related disease, even after successful HIV viral suppression and CD4 count recovery.^2,3,5^ Bowen’s Disease (BD) is a pre-malignant lesion of the skin associated with ultraviolet (UV) light at sun exposed sites, and with HR-HPV and lichen sclerosus at genital sites.^6–8^ Cases of extra-genital cutaneous disease caused by genital HPV genotypes have been reported, including HPV-16 associated BD of the nailbed and fingers.^9–14^

## Case report

A 69-year-old male with well-controlled HIV was referred to Dermatology with a 2-years history of pain, swelling, and nail dystrophy of the right middle finger. He had a past medical history of hyperlipidaemia, transient ischaemia attack, and hiatus hernia. He was diagnosed with HIV in 2010 with baseline HIV viral load of 140,000 copies/ml and nadir CD4 count of 60 cells/mm^3^. After initiation of ART with tenofovir-disoproxil (TDF), emtricitabine, darunavir, and ritonavir, viral suppression was achieved within 5 months and had been maintained since. The CD4 count had reconstituted to 620 cells/mm^3^ within 12 months of ART initiation.

**Figure 1.**
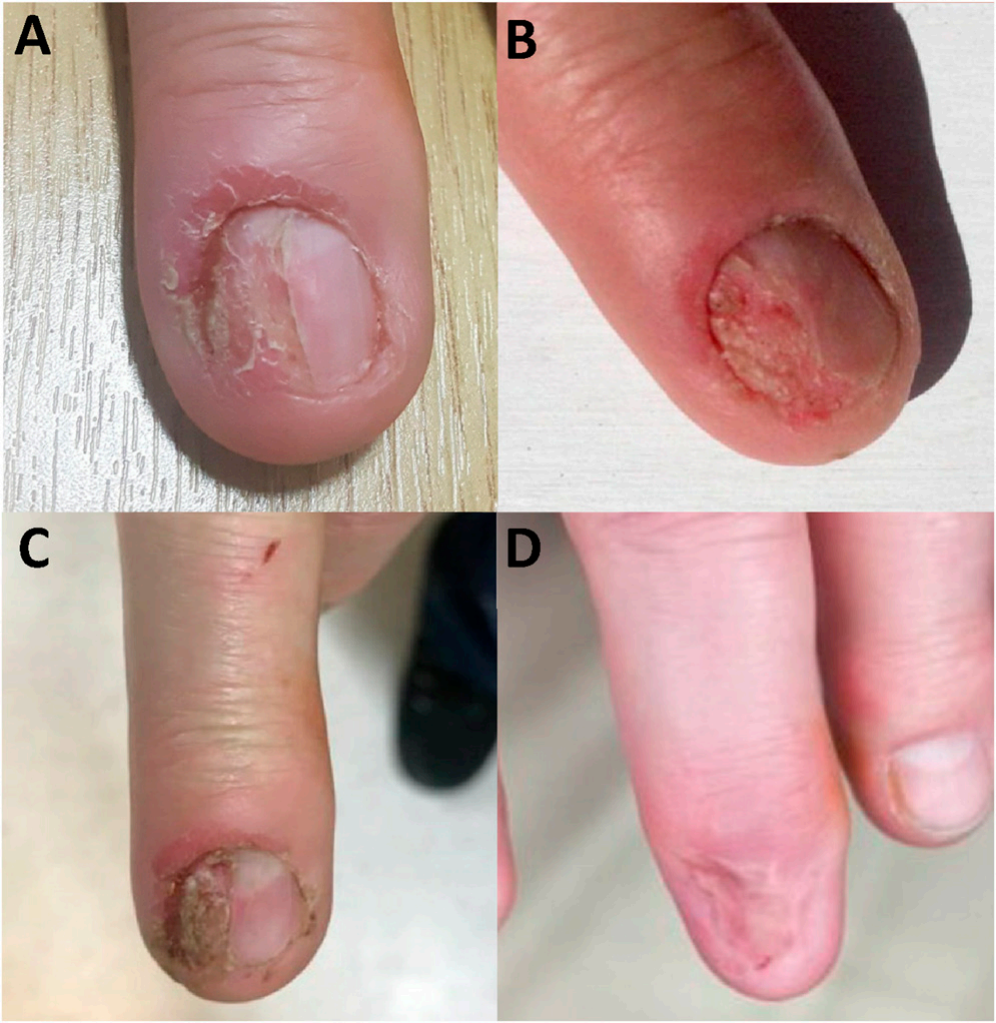
Clinical photographs showing the appearance of the patient’s right middle finger over time: (a) at the time of the patient’s second presentation to dermatology (July 2019), (b) 10 months later and after a diagnostic biopsy had taken place confirming Bowen’s disease due to high-risk HPV (May 2020), (c) following further deterioration of the nail (January 2021), (d) following excision and skin grafting (February 2022).

**Figure 2.**
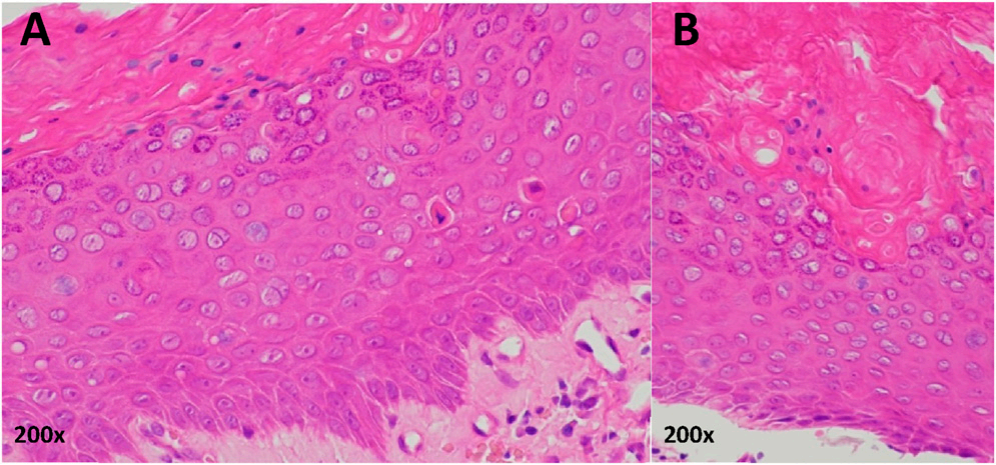
Histology of right middle finger nailbed biopsy (haematoxylin and eosin, October 2019). Two high power views (200x magnification) from a single histologic sample (a) and (b). Sections from the nail biopsy show parakeratosis and irregular acanthosis. There is full-thickness dysplasia of the epidermal keratinocytes and suprabasal mitotic activity amounting to Bowen’s disease.

In June 2016, the patient was seen in the Dermatology clinic with changes to the right middle fingernail and was diagnosed with chronic paronychia. At the time, his CD4 count was 980 cells/mm^3^. He was a smoker with a 10-pack year history. He was prescribed Dermovate® (clobetasol propionate 0.05%), Trimovate® (clobetasone butyrate 0.05%, oxytetracycline 3%, nystatin 100,000units/g), and acetic acid soaks, and was discharged from the service. In July 2019, he was re-referred to Dermatology with a suspected right middle finger nailbed viral wart. He continued to smoke cigarettes. On examination, the right middle fingernail was found to be dystrophic laterally with erythema and swelling of the proximal nail fold (Figure 1). No other fingernails were affected. A biopsy was undertaken, and the histological findings were in keeping with BD of the nailbed (Figure 2). HPV genotyping confirmed the presence of HPV-16.

Following progression of the nailbed disease (Figure 1), in January of 2020 the patient was advised to have the area excised. The patient initially declined excision, and in May of 2021 started a 3-dose schedule of Gardasil-4® post-exposure HPV vaccination. In June of 2021 the patient agreed to proceed with surgery. Excision with full-thickness skin grafting was undertaken; the histology revealed dysplasia extending to the peripheral margins of the sample. A repeat excision was thus undertaken with further histology showing no residual BD. Gardasil-4^®^ HPV vaccination (covering HPV-6, HPV-11, HPV-16, and HPV-18) was completed in December 2021 and at follow-up in February 2022 no residual lesion could be detected (Figure 1). In June 2022, the patient underwent high resolution anoscopy with anal cytology and biopsies, and no evidence of anal intraepithelial neoplasia (AIN) was found. HPV detection was not performed on these samples.

## Discussion

This case adds to a growing body of reported cases on extra-genital manifestations of genital HPV disease. Nailbed HR-HPV disease, particularly in people living with HIV, is an emerging entity in the literature.^9–14^ Any site involved in sexual activity is at risk of infection with ‘anogenital’ HR-HPV strains and may bear the consequences of that. People living with HIV remain at increased risk of HR-HPV associated disease, despite good virological control and immune reconstitution. When assessing atypical cutaneous lesions in people living with HIV, the nadir CD4 count should be considered a potential risk factor for pre-malignancy or malignancy.^3,5^ Alongside historic immunosuppression, smoking was an additional risk factor for HR-HPV disease in this patient, and smoking cessation advice should form part of management for all HPV disease.^6^

Prophylactic HPV vaccination, primarily for girls, was first introduced in 2006 leading to marked reductions in cervical cancer and cervical intraepithelial neoplasia (CIN).^4^ There is also growing evidence and ongoing research on the utility of post-exposure vaccination for HR-HPV disease.^4,6,15^ A meta-analysis of 11 studies found that post-exposure HPV vaccination significantly reduced CIN2/3 recurrences (*n* = 19,909; risk ratio 0.43; 95% confidence interval 0.30-0.60), with recurrences of HPV-16 and HPV-18 associated CIN reduced even further (*n* = 1879; RR 0.26; 95% CI 0.16-0.43).^15^ Whilst prophylactic HPV vaccination is now recommended for all children in the UK, this is not the case in all countries and many adults remain unvaccinated.^16^ Post-exposure HPV vaccination should be considered for all those diagnosed with HR-HPV-related disease.^15^

## References

[1] McBride AA . Human papillomaviruses: diversity, infection and host interactions. Nat Rev Microbiol 2022; 20: 95–108. DOI: 10.1038/s41579-021-00617-5 34522050

[2] Kombe AJ Li B Zahid A , et al. Epidemiology and burden of human papillomavirus and related diseases, molecular pathogenesis, and vaccine evaluation. Front Public Health 2021; 8: 552028. DOI: 10.3389/fpubh.2020.552028 33553082 PMC7855977

[3] Bradbury M Xercavins N García-Jiménez Á , et al. Vaginal intraepithelial neoplasia: clinical presentation, management, and outcomes in relation to HIV infection status. J Low Genit Tract Dis 2019; 23: 7–12. DOI: 10.1097/lgt.0000000000000431 30161052

[4] Kohli M Bunker CB Kravvas G . HPV: an update. Clinics in Dermat 2024. (in press). 10.1016/j.clindermatol.2025.09.012 41005544

[5] Kelly H Chikandiwa A Alemany Vilches L , et al. Association of antiretroviral therapy with anal high-risk human papillomavirus, anal intraepithelial neoplasia, and anal cancer in people living with HIV: a systematic review and meta-analysis. Lancet HIV 2020; 7: e262–e278. DOI: 10.1016/s2352-3018(19)30434-5 32109408

[6] Kravvas G Ge L Ng J , et al. The management of penile intraepithelial neoplasia (PeIN): clinical and histological features and treatment of 345 patients and a review of the literature. J Dermatol Treat 2022; 33: 1047–1062. DOI: 10.1080/09546634.2020.1800574 32705920

[7] Wikström A Hedblad MA Syrjänen S . Penile intraepithelial neoplasia: histopathological evaluation, HPV typing, clinical presentation and treatment. J Eur Acad Dermatol Venereol 2012; 26: 325–330. DOI: 10.1111/j.1468-3083.2011.04069.x 21492254

[8] D’Hauwers KW Depuydt CE Bogers JJ , et al. Human papillomavirus, lichen sclerosus and penile cancer: a study in Belgium. Vaccine 2012; 30: 6573–6577. DOI: 10.1016/j.vaccine.2012.08.034 22939906

[9] Mitsuishi T Sata T Matsukura T , et al. The presence of mucosal human papillomavirus in Bowen’s disease of the hands. Cancer 1997; 79: 1911–1917. DOI: 10.1002/(sici)1097-0142(19970515)79:10<1911::aid-cncr11>3.0.co;2-y 9149017

[10] McGrae JD Jr. Greer CE Manos MM . Multiple Bowen's disease of the fingers associated with human papilloma virus type 16. Int J Dermatol 1993; 32: 104–107. DOI: 10.1111/j.1365-4362.1993.tb01446.x 8382665

[11] Shimizu A Kuriyama Y Hasegawa M , et al. Nail squamous cell carcinoma: a hidden high-risk human papillomavirus reservoir for sexually transmitted infections. J Am Acad Dermatol 2019; 81: 1358–1370. DOI: 10.1016/j.jaad.2019.03.070 30930083

[12] Turowski CB Ross AS Cusack CA . Human papillomavirus-associated squamous cell carcinoma of the nail bed in African-American patients. Int J Dermatol 2009; 48: 117–120. DOI: 10.1111/j.1365-4632.2009.03450.x 19200182

[13] Namgoong S Kim J Jeong KM , et al. Association of human papillomavirus and extra-genital Bowen disease (squamous cell carcinoma in situ): a systematic review. J Am Acad Dermatol 2021; 84: 822–825. DOI: 10.1016/j.jaad.2020.09.059 33010321

[14] Montesu MA Onnis G Lissia A , et al. Extragenital human papillomavirus 16-associated Bowen's disease. Indian J Dermatol 2017; 62: 97–98. DOI: 10.4103/0019-5154.198035 28216736 PMC5286765

[15] Kechagias KS Kalliala I Bowden SJ , et al. Role of human papillomavirus (HPV) vaccination on HPV infection and recurrence of HPV related disease after local surgical treatment: systematic review and meta-analysis. BMJ 2022; 378: e070135. DOI: 10.1136/bmj-2022-070135 35922074 PMC9347010

[16] World Health Organization . Human papillomavirus vaccine: WHO position paper 2022; 97: 645–672. Available at https://www.who.int/publications/i/item/who-wer9750-645-672 [accessed 31/01/2024]

